# Cumulative evidence for relationships between multiple variants in 8q24 and colorectal cancer incidence

**DOI:** 10.1097/MD.0000000000011990

**Published:** 2018-08-21

**Authors:** Yu Tong, Huiqing Wang, Shiping Li, Fengyan Zhao, Junjie Ying, Yi Qu, Dezhi Mu

**Affiliations:** a Department of Pediatrics; b Key Laboratory of Obstetric & Gynecologic and Pediatric Diseases and Birth Defects of Ministry of Education, West China Second University Hospital, Sichuan University, Chengdu, Sichuan Province, China.

**Keywords:** 8q24, colorectal cancer, genetic variant, susceptibility

## Abstract

Genome-wide association studies (GWAS) have identified multiple independent cancer susceptibility loci at chromosome 8q24.

We conducted a comprehensive research synopsis and meta-analysis to evaluate associations between 6 variants in 8q24 and risk of colorectal cancer using data from 31 eligible articles totaling 41,942 cases and 49,968 controls.

Of the 6 variants located in 8q24, 3 were significantly associated with risk of colorectal cancer. In particular, both homozygous TT and heterozygous CT genotypes of rs10505477, as well as the GG and TG genotypes of rs6983267, were associated with risk of colorectal cancer.

Our study provides summary evidence that common variants in the 8q24 are associated with risk of colorectal cancer in this large-scale research synopsis and meta-analysis. Further studies are needed to explore the exact role of the variants in the 8q24 involved in the etiology of colorectal cancer.

## 1 Introduction

Colorectal cancer (CRC) is the third leading cause of cancer-related mortality worldwide. Many influencing factors are associated with the risk of CRC. Among the risk factors and causes for CRC, inherited genetic factors account for approximately 35% of the disease etiology.^[1]^ In the past few years, several genome-wide association studies (GWAS) have identified novel loci that are associated with CRC risk, including variants on8q24, 8q23.3, 10p14, 11q23, 15q13, 18q21, and so on.^[2,3]^

Variants on 8q24 have shown strong evidence of an association with the risk of CRC in different populations. The human c-myc gene is located at 8q24 on the long arm of chromosome 8. Variant rs6983267 was firstly identified to be significantly associated with colorectal cancer.^[1]^ In 2007, Tomlinson and colleagues^[4]^ conducted a genome-wide association study of 550,000 tag SNPs (single nucleotide polymorphisms) in 930 familial colorectal tumor cases and 960 controls and found that the most strongly associated SNP was rs6983267. In the same year, Poynter et al^[5]^ conducted a case-unaffected sibling analysis using population- and clinic-based discordant sibships to investigate the associations between common variants at 8q24 and risk of CRC, and detected statistically significant associations between rs6983267 and rs10505477 on 8q24 and risk of CRC. More recently, long noncoding RNAs (lncRNAs) originated from the 8q24 region show relevance with multiple types of cancers. A large proportion of these lncRNAs that surrounds the essential Wnt target *MYC* gene, show significant association with CRC incidence, the extent of malignancy, and patient prognosis.^[6]^*CAT1-S*, known as *CARLo-5*, is upregulated in premalignant conditions during CRC transformation. Knockdown of *CCAT1-S* decreased CRC cell growth in vitro and in vivo. Interestingly, the expression of *CCAT1-S* is significantly correlated with the allele status of the SNP rs6983267. Further study demonstrated that the rs6983267-containing region interact with *CCAT1-S* promoter and regulate its expression.^[7]^ Based on the above compelling evidence, it was hypothesized that the genetic variants in the 8q24 region played important roles in colorectal carcinogenesis.

In the present study, we performed a comprehensive meta-analysis, involving a total of 41,942 cases and 49,968 controls, to evaluate all genetic studies that investigated associations between 6 variants in the 8q24 region and risk of colorectal cancer.

## 2 Methods

All methods were based on guidelines proposed by the Human Genome Epidemiology Network for systematic review of genetic association studies and followed the guidelines of Preferred Reporting Items for Systematic Reviews and Meta-Analyses. As it is a meta-analysis of the previous works of literature, approval of the ethics committee was not required.

### 2.1 Search strategy and selection criteria

We systematically searched PubMed and Embase to identify genetic association studies published in print or online before February 8th, 2018 in English language using key terms “8q24” and “variant or polymorphism or genotype” and “colorectal cancer or colorectal carcinoma or colorectal tumor.” The eligibility of each study was assessed independently by 2 investigators (YT and HW). The articles included in the meta-analysis must meet the following inclusion criteria: evaluating the associations of genetic variants in the 8q24 with risk of colorectal cancer; providing age-adjusted or multivariate-adjusted risk estimates (e.g., relative risks [RRs], hazard ratios [HRs], odds ratios [ORs], 95% confidence intervals [CIs] or standard errors (SEs) or sufficient data to calculate these estimates). Studies were excluded when: they lacked sufficient information; they were not published as full reports, such as conference abstracts and letters to editors; and they were studies of cancer mortality (rather than incidence).

### 2.2 Data extraction

Data were extracted by 2 investigators (YT and HW), who used recommended guidelines for reporting on meta-analyses of observational studies. Data extracted from each eligible publication included first author, publishing year, study design, method of case selection, source population, ethnicity of participants, sample size, variants, major and minor alleles, genotype counts for cases and controls, Hardy–Weinberg equilibrium (HWE) among controls. Ethnicity was classified as African (African descent), Asian (East Asian descent), Caucasian (European descent), or other (including Native Hawaiians, Latinos, etc.) based on the ethnicity of at least 80% of the study population. In total, 31 eligible publications had sufficient data available for extraction and inclusion in meta-analyses.

### 2.3 Statistical analysis and assessment of cumulative evidence

The odds ratio was used as the metric of choice for each study. To detect overall genetic associations, allele frequencies were computed for studies reporting allele and genotype data. Pooled odds ratios were computed by the fixed effects model and the random effects model based on heterogeneity estimates. Once an overall gene effect was confirmed, the genetic effects and mode of inheritance were estimated using the genetic model-free approach suggested by Minelli et al. We performed Cochran's *Q* test and calculated *І*^2^ statistic to evaluate heterogeneity between studies. *І*^2^ values < 25% represent no or little heterogeneity, values 25% to 50% represent moderate heterogeneity, and values > 50% represent large heterogeneity. Sensitivity analyses were conducted to examine if the significant association would be lost when the first published report was excluded, or studies deviated from HWE in controls were excluded. Harbord's test was performed to evaluate publication bias. Small study bias was calculated by egger's test. All analyses were conducted using Stata, version 14.0 (StataCorp, College Station, TX, 2017), with the *metan, metabias, metacum, and metareg*commands.

## 3 Results

### 3.1 Eligible studies

**Figure 1:**
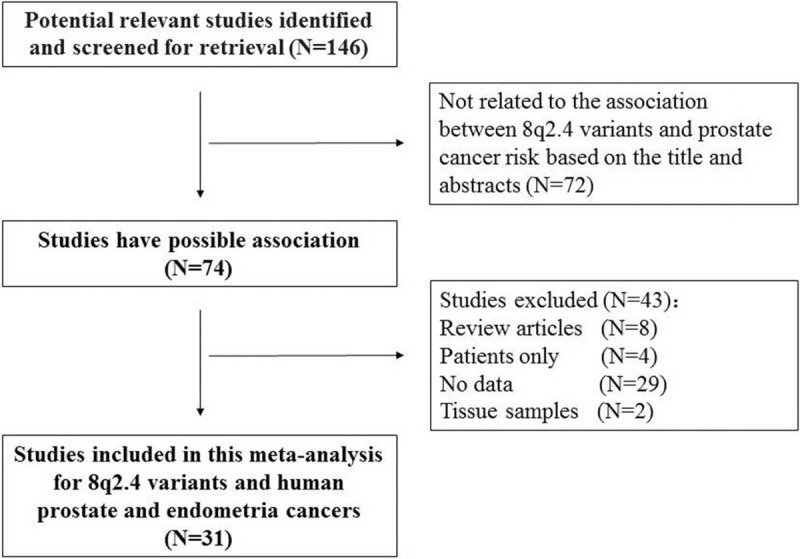
Flow diagram of included and excluded studies.

Our initial database search identified 146 potentially relevant studies. Based on a review of titles and abstracts, 74 articles were retained. The full text of these 74 articles was reviewed in detail, and 31 studies were eligible for inclusion in the meta-analysis. The specific process for identifying eligible studies and inclusion and exclusion criteria are summarized in Figure 1.

### 3.2 Allelic associations

Of the 6 variants located in 8q24, 3 were significantly associated with risk of colorectal cancer, including rs10505477, rs6983267, and rs10808556. No significant associations were found between rs1447295, rs7837328, and rs10090154 and colorectal cancer (data not shown).

#### 3.2.1 rs10505477 C > T

**Figure 2:**
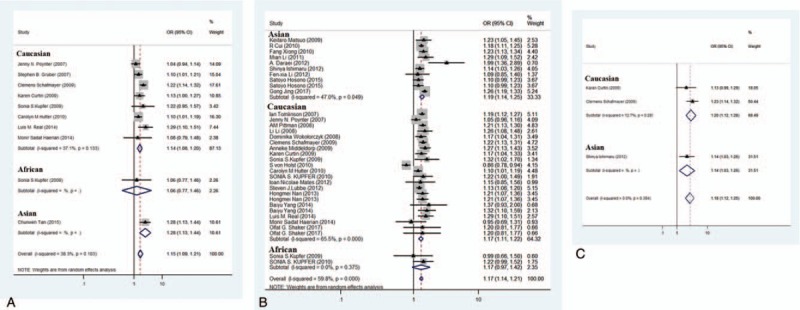
Forest plots for associations between selected variants in the 8q24 region and colorectal cancer risk. Associations of rs10505477 (A), rs6983267 (B), and rs10808556 (C).

Nine studies were included (Table 1), and a significant association with risk of colorectal cancer was found (*P = *6.66 × 10^−8^, random effect OR = 1.15, 95% CI: 1.09, 1.21; *Q* = 14.58, *P = *.103, *I*^*2*^ = 38.3%, Fig. 2A). A similar pattern was observed for Caucasians (*P = *6.48 × 10^−6^, random effect OR = 1.14, 95% CI: 1.08, 1.20; *Q* = 11.13, *P = *.133, *I*^2^ = 37.1%). No publication bias was found in the eligible studies (Harbord's test *P = *.840, Table 2).

**Table 1:**
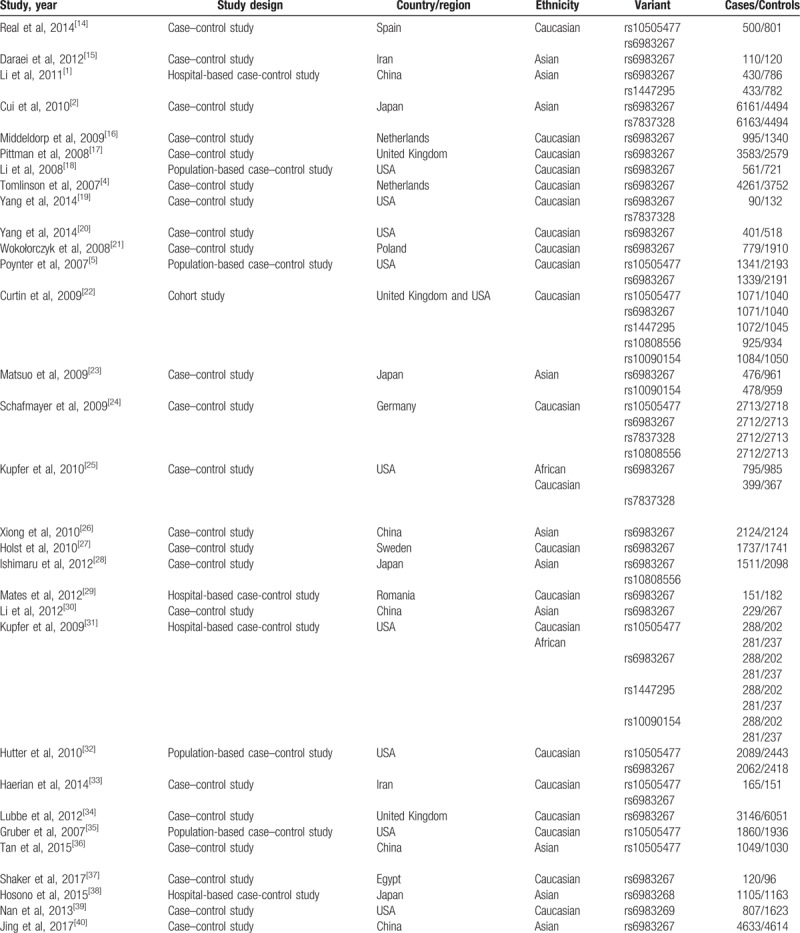
Characteristics of the included articles.

**Table 2:**
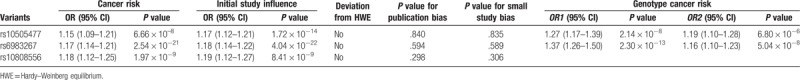
Details of genetic variants significantly associated with cancer risk in meta-analyses.

#### 3.2.2 rs6983267 T > G

Twenty-nine studies were included (Table 1), and a significant association with risk of colorectal cancer was found (*P = *2.54 × 10^−21^, random effect OR = 1.17, 95% CI: 1.14, 1.21; *Q* = 82.00, *P = *.000, *I*^*2*^ = 59.8%, Fig. 2B). Significant association was also found for Asians (*P = *1.71 × 10^−13^, random effect OR = 1.19, 95% CI: 1.14, 1.25; *Q* = 16.99, *P = *.049, *I*^2^ = 47.0%) and Caucasians (*P = *4.40 × 10^−11^, random effect OR = 1.17, 95% CI: 1.11, 1.22; *Q* = 60.82, *P = *.00, *I*^2^ = 65.5%). No publication bias was found in the eligible studies (Harbord's test *P = *.594, Table 2).

#### 3.2.3 rs10808556T > C

Three studies were included (Table 1), and a significant association with risk of colorectal cancer was found (*P = *1.97 × 10^−9^, fixed effect OR = 1.18, 95% CI: 1.12, 1.25; *Q* = 1.86, *P = *.394, *I*^*2*^ = 0.0%, Fig. 2C). No publication bias was found in the eligible studies (Harbord's test *P = *.298, Table 2).

### 3.3 Genotype comparison

#### 3.3.1 rs10505477 C > T

Of the 9 studies, 5 reported genotype information. The genotype effects for TT versus CC (*OR*1) and CT versus CC (*OR*2) were calculated for each study. A multivariate meta-analysis was conducted to estimate the pooled risk (Table 2). There was a significantly increased risk of colorectal cancer among individuals with the homozygous TT genotype (*P = *2.14 × 10^−8^, random effect *OR*1 = 1.27, 95% CI: 1.17, 1.39; *Q* = 7.44, *P = *.115, *I*^2^ = 46.2%) and heterozygous CT genotype (*P = *6.80 × 10^−6^, random effect *OR*2 = 1.19, 95% CI: 1.10, 1.28; *Q* = 3.77, *P = *.438, *I*^2^ = 0.0%).

#### 3.3.2 rs6983267 T > G

Of the 29 studies, 21 reported genotype information. The genotype effects for GG versus TT (*OR*1) and TG versus TT (*OR*2) were calculated for each study. A multivariate meta-analysis was conducted to estimate the pooled risk (Table 2). There was a significantly increased risk of colorectal cancer among individuals with the homozygous GG genotype (*P = *2.30 × 10^−13^, random effect *OR*1 = 1.37, 95% CI: 1.26, 1.50; *Q* = 69.80, *P = *.000, *I*^2^ = 67.0%) and heterozygous TG genotype (*P = *5.04 × 10^−8^, random effect *OR*2 = 1.16, 95% CI: 1.10, 1.23; *Q* = 41.95, *P = *.009, *I*^2^ = 45.2%).

### 3.4 Sensitivity analysis

Sensitivity analysis for the results of 8q24 variants and colorectal cancer risk demonstrated that the obtained results were statistically robust and no individual study affected the pooled OR significantly (Table 2).

## 4 Discussion

To our knowledge, this study is the largest and most comprehensive assessment of literatures on associations between genetic variants in the 8q24 region and colorectal cancer risk. Preliminary meta-analyses were mostly limited to single or less SNPs in relation to colorectal cancer. Here we performed a research synopsis and meta-analysis to systematically evaluate associations between 6 variants in 8q24 region and risk of colorectal cancer using data from 31 articles totaling 41,942 cases and 49,968 controls. Our study not only provides an update of the variants analyzed previously, but also evaluates more variants that have not been analyzed in previous meta-analyses.

Of the 6 variants located in 8q24, 3 were significantly associated with risk of colorectal cancer. Our primary analysis shows that, the rs10505477 (*P = *1.08 × 10^−12^, OR = 1.48), rs6983267 (*P = *2.54 × 10^−21^, OR = 1.17), rs10808556 (*P = *1.97 × 10^−9^, OR = 1.18) were significantly associated with risk of colorectal cancer. In particular, both homozygous TT (*P = *2.14 × 10^−8^, *OR*1 = 1.27) and heterozygous CT (*P = *6.80 × 10^−6^, *OR*2 = 1.19) genotypes of rs10505477, as well as the GG (*P = *2.30 × 10^−13^, *OR*1 = 1.37) and TG (*P = *5.04 × 10^−8^, *OR*2 = 1.16) genotypes of rs6983267, were associated with risk of colorectal cancer. Our findings were based on several gene-association studies, including several thousand participants, and were robust in terms of study design and sensitivity analyses. We found no evidence of publication bias or small study bias based on funnel plots. Using data from Phase 3 of the 1000 Genomes Project,^[8]^ we found thatrs6983267is in strong LD with both the rs10505477 and the rs10808556 in Europeans and Asians (*r*^2^ > 0.05 for all tests), whereas is in weak LD (*r*^2^ < 0.05 for all tests) in Africans. These findings suggest that variants may be distinct in different ethnic groups.

Multiple variants have been identified to be correlated with CRC risk. These variants might be involved in signaling pathway, and lead to higher CRC risk subsequently.^[9,10]^ The 8q24 region is a desert with multiple SNPs associated with CRC risk. More recently, this region was proposed as a typical transcriptional super-enhancer binding directly to DNA sequence motifs, which are required at key oncogenes and at genes that function in the acquisition of hallmark capabilities in cancer.^[11]^ In addition, various enhancer activities were affected by these SNPs. The alleles of rs6983267, differentially bind transcription factor 7-like 2 and physically interacts with the *MYC* proto-oncogene.^[12]^ Another study found that rs6983267 also affects binding of the Wnt-regulated transcription factor TCF4 in a regulatory element, with the risk allele G showing stronger binding that is functional in CRC cells.^[13]^ These data provide strong support for a biological mechanism underlying 8q24 variants in genesis of colonic neoplasia.

## 5 Conclusion

Our study provides evidence that common 3 variants in the 8q24 region are associated with risk of CRC. Further functional studies are needed to explore the exact mechanisms of 8q24 variants involved in parthenogenesis of colorectal cancer.

## Author contributions

Data were extracted independently by Yu Tong and Huiqing Wang, Yu Tong, Shiping Li, Fengyan Zhao, and Dezhi Mu contributed to writing the manuscript. Data with any disagreement was adjudicated by Yi Qu.

**Data curation:** Shiping Li.

**Investigation:** Huiqing Wang, Fengyan Zhao, Junjie Ying.

**Software:** Yu Tong.

**Writing – original draft:** Yi Qu.

**Writing – review & editing:** Dezhi Mu.

Dezhi Mu orcid: 0000-0002-2599-7041
